# Vascular Mechanobiology: Towards Control of In Situ Regeneration

**DOI:** 10.3390/cells6030019

**Published:** 2017-07-03

**Authors:** Eline E. van Haaften, Carlijn V. C. Bouten, Nicholas A. Kurniawan

**Affiliations:** 1 Department of Biomedical Engineering, Eindhoven University of Technology, P.O. Box 513, 5600 MB Eindhoven, The Netherlands; e.e.v.haaften@tue.nl (E.E.v.H.); c.v.c.bouten@tue.nl (C.V.C.B.); 2 Institute for Complex Molecular Systems, Eindhoven University of Technology, P.O. Box 513, 5600 MB Eindhoven, The Netherlands

**Keywords:** in situ tissue engineering, regeneration, vessels, mechanotransduction, mechanosensing, growth and remodeling, tissue homeostasis, scaffolds

## Abstract

The paradigm of regenerative medicine has recently shifted from in vitro to in situ tissue engineering: implanting a cell-free, biodegradable, off-the-shelf available scaffold and inducing the development of functional tissue by utilizing the regenerative potential of the body itself. This approach offers a prospect of not only alleviating the clinical demand for autologous vessels but also circumventing the current challenges with synthetic grafts. In order to move towards a hypothesis-driven engineering approach, we review three crucial aspects that need to be taken into account when regenerating vessels: (1) the structure-function relation for attaining mechanical homeostasis of vascular tissues; (2) the environmental cues governing cell function; and (3) the available experimental platforms to test instructive scaffolds for in situ tissue engineering. The understanding of cellular responses to environmental cues leads to the development of computational models to predict tissue formation and maturation, which are validated using experimental platforms recapitulating the (patho)physiological micro-environment. With the current advances, a progressive shift is anticipated towards a rational and effective approach of building instructive scaffolds for in situ vascular tissue regeneration.

## 1. Introduction

Cardiovascular diseases are the leading cause of death globally. While pharmacological interventions can help slow down the progress of various cardiovascular diseases, the eventual outcome is often chronic tissue degeneration or damage and loss of function. Solving this problem traditionally requires whole organ/tissue removal and replacement (i.e., heart, valve, or vessel), thereby generating an enormous clinical demand for organ and tissue availability, which is unmet. For example, the most commonly-employed clinical solution for a heart attack caused by atherosclerosis is grafting of autologous arteries and veins to bypass blocked and damaged vessels; however, vessel availability is scarce due to diseases and repeated harvesting, and autologous harvest has been associated with considerable morbidity [1,2]. Synthetic, tissue-engineered vascular substitutes have been developed to alleviate this shortage by offering the prospect of a functional graft and vessel as alternates to native vein or artery for vascular regeneration, but this approach also has its drawbacks. Cell-laden constructs and hydrogels require labor-intensive, lengthy, and expensive cell isolation and in vitro culture steps, whereas failure of cell engraftment and long-term cell and material survival remain a frequent issue with cell-free scaffolds and decellularized tissues [3,4]. Small-diameter (<6 mm) synthetic applications are especially prone to thrombosis and failure [5]. Another pressing problem is that conventional, non-degradable tissue-engineered constructs often fail to adapt to changes in the host, for example as a result of disease, growth, and aging. This limitation necessitates multiple follow-up procedures that significantly reduce the quality of life and at the same time multiply the costs for the patient.

At the heart of these current challenges is our lack of knowledge about how cells respond to the new and sometimes harsh vascular micro-environment that is being presented to them. Specifically, our understanding of the in vivo cell-mediated tissue remodeling process is still rudimentary, restricting the nature of our efforts in regenerating functional tissues largely to observations or trial-and-error. A key step to move towards a more hypothesis-driven engineering approach, therefore, is to obtain a better understanding of cell/tissue response and remodeling in (patho)physiological situations. In fact, this step goes hand in hand with the emergence of a relatively new paradigm in regenerative medicine: in situ tissue engineering (TE). The idea of this approach is to fully exploit the body’s regenerative capacity and use cell-free, readily-available bioresorbable scaffolds that are designed to recruit the desired cells and promote neotissue formation directly in the host’s functional site. Indeed, in vitro, in vivo, and in situ approaches for engineering vascular grafts have been embraced and developed [6,7]. In vitro TE requires an extended culturing time including mechanical preconditioning in the lab prior to implantation. Examples exploiting this approach are Niklason’s biodegradable, scaffold-based decellularized graft [8] and L’Heureux’s cell self-assembled vessel [9]. In vivo TE uses the ‘body-as-bioreactor’: a tubular template is implanted in the peritoneal cavity or subcutaneous pouch, around which a tubular capsule is allowed to form, which can be harvested as a graft [10]. Despite these efforts, in situ TE remains clinically the most appealing option because of its off-the-shelf availability and immediate functionality. Both in vitro and in situ TE vascular grafts have shown their applicability in human clinical trials [7], with positive results under pulmonary pressures and with large vessel diameters (ϕ> 6 mm) [11].

As we shall discuss in this article, an important lesson that has been recognised from these studies is the crucial role of mechanics (and the underlying tissue macro- and microstructures) not only in maintaining tissue’s mechanical integrity/strength, but also in governing the vessel’s response to hemodynamics loading, in directing the cellular organization and activity within the tissue, as well as in stimulating the matrix formation and remodeling in response to physiological or pathological stimuli. Fortunately, mechanobiology-oriented research in the past decade has shed much light on the mechanosensitivity of virtually all vascular cells (e.g., fibroblasts, endothelial cells, smooth muscle cells). This growing body of knowledge paves the way for a smarter design of instructive scaffolds that can actively direct cell behavior and facilitate the formation of a new functional tissue, and hence advances ongoing efforts to develop regenerative medicine approaches to treat cardiovascular diseases.

In this review, we focus on three crucial aspects that need to be taken into account in translating the fundamental mechanobiological insights into the regeneration of vessels. Specifically, we limit our present discussions to relatively thick-walled blood vessels (i.e., arteries and, to some extent, veins), which are accessible to in situ TE approaches and where principles of cellular mechanobiology offer exciting new possibilities. First, we discuss the structure–function relation of vascular tissues and how it strongly contributes to the attaining of tissue mechanical homeostasis. Second, we summarize the main mechanobiological cues that have been identified to govern cell functions, which are present in vascular tissues. Lastly, we review the requirements for building an instructive scaffold for in situ cardiovascular TE and the available experimental platforms to test its response to (patho)physiological conditions.

## 2. Mechanosensing to Mechanical Homeostasis

Tissues, including vessels, are remarkably well-adapted to the specific mechanical demands expected from them. This mechanical stability, e.g., the preferred amounts of wall stresses and strains in a blood vessel, is referred to as mechanical homeostasis and is thought to be the result of growth and remodeling processes. A homeostatic state at the tissue level can be maintained by mass deposition and mass degradation, i.e., via cell-mediated growth and remodeling, occurring at the timescale of weeks to years. Crucially, the newly-formed tissue must be deposited with a certain organization and pre-stress in order to maintain the overall structure and function during matrix turnover [12]. This is an especially important consideration for in situ TE approaches, where the tissue/scaffold graft needs to be functional not only directly at the time of implantation, but also throughout the neotissue formation and maturation. In this section, we discuss the interrelationship between function and structure of blood vessels. Starting from a tissue level, we further elaborate on the possible mechanisms that can maintain this relationship across multiple scales, down to the cellular level.

### 2.1. Mechanical Behavior of Blood Vessels

In order for a blood vessel to fulfill its primary function, i.e., blood transportation, a tubular geometry is required with sufficient distensibility and strength. The mechanical performance, i.e., distensibility and strength, of vascular prostheses is usually evaluated against native tissues in terms of burst pressure, suture retention strength, and compliance. Among other things, these parameters are used to predict potential clinical utility.

Compliance—a measure for stiffness—of arteries is estimated to be in the range of 3–12%/100 mmHg under physiological pressures (80–120 mmHg), but strongly depends on species and location (Table 1). Compared to native tissues, synthetic grafts tend to be less compliant. Even though synthetic grafts initially appear too stiff, after in situ tissue formation and graft degradation, the compliances are well within the range for native arteries (Table 1).

However, one should be cautious with grafts that degrade too quickly [13], which can risk the formation of aneurysms [29] or even wall rupture. In contrast to synthetic grafts, in vitro tissue-engineered blood vessels exhibit compliances close to native values (Table 1), although still in the lower range. Burst pressure—a measure for strength—ranges between 2000 mmHg and 3000 mmHg in arteries (Table 1). Similar to the case for compliance, in situ and in vitro tissue-engineered grafts outperform synthetic grafts with respect to burst pressure, although with subtler differences compared to compliance.

The lower compliance of the scaffold compared to native tissue is considered to be one of the underlying problems for graft failure. To illustrate, with closer agreement of compliance between the graft and its surrounding vessel, graft patency has been found to increase up to 75%. Interestingly, compliance and patency has been shown to be highly significantly correlated [30]. The mechanism behind the development of stenosis in low-compliant grafts might be attributed to inward growth and remodeling (Section 2) in response to increased circumferential stresses or changes in blood flow (Section 3.2).

The mechanical behavior of arteries, i.e., compliance and burst pressure, is attributable to the composition and structure of its main components: collagen, elastin, and smooth muscle. This means that a vessel relies on mechanisms of growth and remodeling that regulate tissue composition and tissue structure to maintain its primary function.

### 2.2. Microstructure of Blood Vessels

**Figure 1:**
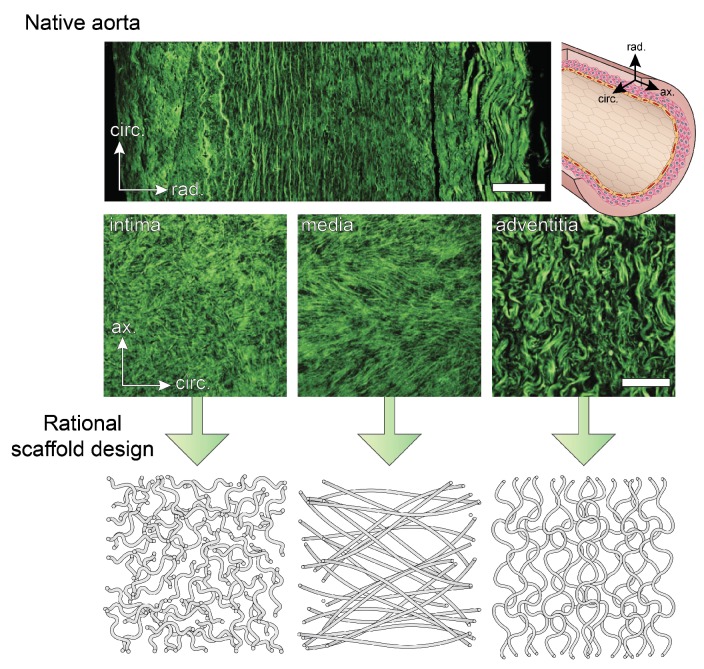
Collagen microstructure of the three different layers of the aortic wall visualized with second harmonic generation microscopy (reproduced with permission from [33]). Mimicking the native collagen organization in fibrous scaffolds should be one of the strategies in the design of instructive scaffolds. Scalebar = 100 μm.

The different layers of a healthy blood vessel wall are distinguishable according to their collagen microstructure, which is strongly correlated with stiffness and strength. Typically, the collagen microstructure is visualized with second harmonic generation (SHG) microscopy or with fluorescently labeled collagen using CNA35 [31], sometimes in combination with optical clearing [32]. From these measurements, the intima is recognized as a layer with highly dispersed fibres around the circumferential direction, whereas the characteristic features of the media are the two fibre bundles at an angle of about 30° with respect to the circumference. The adventitia contains thicker, tortuous fibres that are mainly axially aligned [33] (Figure 1). However, variations on the collagen structure exist in different regions of the arterial tree. For example, unlike the typical net-like structure found in most arteries, the iliac artery contains fibres parallel to the circumference [34].

**Figure 2:**
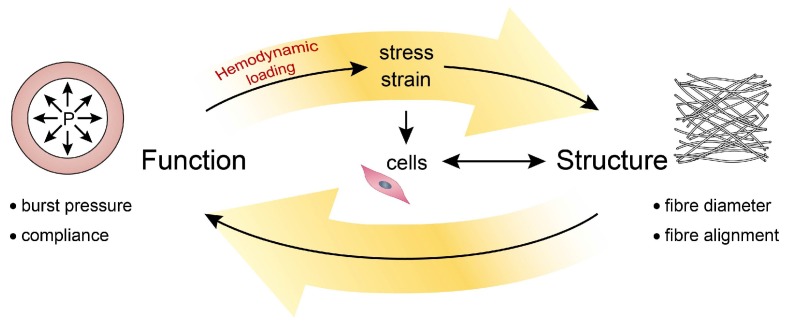
Interrelationships between structure and function: functionality (in terms of mechanical properties) follows directly from structure, which is regulated by cell-mediated growth and remodeling in response to mechanical loading (in terms of stress and strain).

The underlying explanation for the differences in fibre orientation is found in the different magnitudes of axial pre-stretch: using computational modeling, the relatively low axial pre-stretch in the iliac artery was found to be directly associated with a single fibre bundle [35]. These findings highlight the interrelationship between mechanics and structure: the mechanical properties of the blood vessel wall determine how hemodynamic loading is translated into stresses and strains, such as residual stress. In turn, these stresses and strains dictate structure either directly (i.e., passively), or through cell-mediated growth and remodeling (i.e., actively) (Figure 2).

The functional performance of engineered tissues is likely to become comparable to that of biological vessels when efforts are focused on mimicking the compositions and structures of native tissues. In a study comparing the collagen ultrastructure between native and dynamically-strained engineered arteries, it was found that engineered collagen fibres are thinner, more loosely packed, and aligned more axially rather than circumferentially [36]. The differences in alignment may partly be explained by the initial microstructure of the scaffold, which contained relatively more axial fibres than circumferential fibres (i.e., a contact-guidance effect) [36]. Again, this suggests the importance of focusing on fine-tuning the scaffold’s microstructure. Mimicking the native collagen structure in fibrous scaffolds, which is typically anisotropic, is the first step. Using such anisotropic scaffolds for in situ TE improves the initial mechanical performance of the graft, guides tissue formation in the desired direction in the early stages [37], and allows for sufficient tissue maturation in the long-term.

### 2.3. Maintaining Mechanics and Microstructure: Mechanical Homeostasis

In situ TE relies on the principles of growth and remodeling to preserve functionality during and after the different regeneration stages, from the time of implantation until a viable construct is achieved. The potential of this approach has been demonstrated by growing animal models, where tubular scaffolds increase in diameter and develop tissue similar to native tissue [38], although the relative contributions from somatic growth and plastic deformation often remains to be elucidated. Predicting growth, i.e., increase in cell volume, cell number, and/or ECM, in computational models can help us in the design of new scaffolds. This requires an identification of tissue-specific growth laws which are generally anisotropic for cardiovascular tissues [39] meaning that the growth rate is not equal in every direction. Remodeling is a term that is usually used in combination with growth, and is attributed to scenarios where a change in properties, such as anisotropy, stiffness, and strength, is observed in time, usually in response to hemodynamic loading [40]. These changes are manifestations of changes in the underlying microstructure, which may be accompanied by growth as well [41].

**Figure 3:**
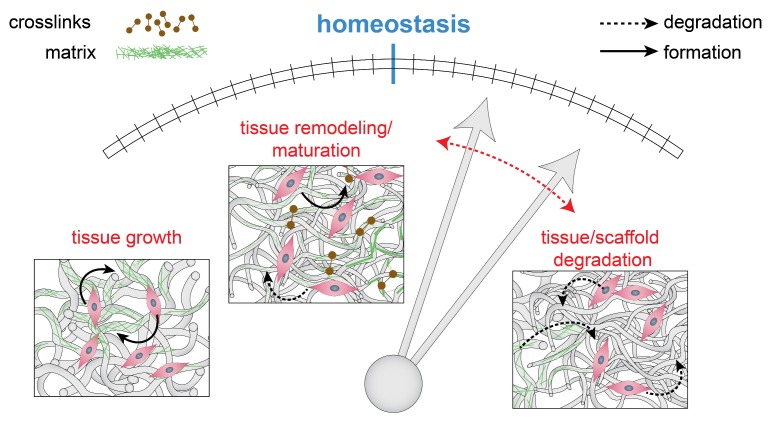
Mechanical homeostasis is a delicate balance between tissue growth, degradation, and remodeling. A perturbation of the equilibrium will result in a relative upregulation of one of these processes (indicated in red), in an attempt to restore the balance.

Out-of-equilibrium cases, i.e., perturbation of the mechanical homeostatic state, can provide us with new information about the underlying principles of maintaining homeostasis (Figure 3). If the magnitude of a perturbation is too large, e.g., large increase in pressure or flow, it is unlikely that a vessel wall is able to restore homeostasis. In those cases, a tissue is said to be mechanobiologically unstable, where geometry and functionality cannot be maintained in the long-term [42]. By inducing smaller changes, e.g., small increments in pressure, the adaptation process is more likely to restore equilibrium [43]. Here, the tissue is in a mechanobiological stable state where perturbations remain small or reach zero [42].

To give an example of this phenomenon, consider the homeostatic state for fluid flow, which is hypothesized to be a streamlined flow. In contrast to stable flow, flow disturbances give rise to platelet activation, and proliferation and migration of vascular smooth muscle cells, leading to intimal hyperplasia (IH) and thrombosis. Such flow disturbances occur for example near the anastomotic border of an arteriovenous graft, which is clinically found to occlude in many cases [44]. Hence, rationally designing vascular grafts should include minimizing flow disturbances [43].

It is important to note that the magnitude of the initial perturbation does not uniquely determine the tissue’s adaptive response. Some tissues enter a self-reinforcing process, which is maintained by growth and remodeling. In mechanobiologically unstable vessels, even small perturbations can lead to progressive changes in the long-term. In fact, mechanobiological instability is hypothesized to be the initiation mechanism of aneurysms, i.e., local dilations of the vessel wall that can still be mechanically stable. For example, age-related vessel dilations and aneurysms after perturbation of pressure or loss of elastin can indeed be predicted by incorporating the theoretical basis of growth and remodeling in a computational framework [42]. Similarly, it has been shown that, with its limited adaptive capacity, a vein can adapt to only small perturbations in blood pressure [45]. As another example, biomechanical diversity among implanted vascular grafts can be computationally explained by the variations in the ratio of collagen type I/III, which was found to be supported by experiments [46] (Table 2). Hence, computational frameworks are very valuable for understanding pathological cases, predicting mechanobiological (in)stabilities in (engineered) tissue, and thus in guiding new scaffold designs.

Although such computational models provide insight in the role of ECM turnover in mechanical homeostasis at a tissue/organ level, they usually do not include the cues that guide cells to deposit, rearrange, or remove ECM. In order to understand cell-mediated growth and remodeling, a translational approach that converts classical biomechanics to single cell mechanics—and vice versa— is required, i.e., multi-scale computational modeling. In line with this, it has been suggested that mechanical homeostasis exists across multiple levels of organization, from tissue to cellular and sub-cellular levels [49,50]. For example, the tensional homeostasis which is suggested to be present at a tissue level is also found at a cellular level: fibroblasts change their cytoskeletal configuration in order to maintain their own stress state [51,52]. One of the consequences of tensional homeostasis is that it encompasses a mechanism to control ECM and cellular stiffness: due to the strain-stiffening behavior of most soft tissues and their cellular cytoskeleton, stiffness is proportional to stress. This means that cellular control of stress is equivalent to cellular control of stiffness.

## 3. Passive and Active Cues in the Vessel

These crucial roles of cellular adaptation in defining tissue microstructure and its ability to achieve mechanical homeostasis raise an important question: what are the physiologically-relevant mechanical situations that need to be recapitulated in in situ TE? Physiological mechanical cues can be broadly categorized into ‘passive’ cues—ones that simply define the physical environment in which the cells reside–and ‘active’ cues—ones that directly provoke cell response by actively stimulating the cells. Passive cues, such as tissue/scaffold morphology and mechanical properties, are sensed by the cells through active (or inside-out) mechanosensing action. Here, cells actively expend energy to generate forces necessary to probe their physical environment, akin to one testing the firmness of a tomato in a supermarket by squeezing it. As one then decides whether or not to buy the tomato based on its mechanical properties, cells also make important decisions whether to reorient, migrate, or differentiate based on their perception of the passive cues provided by their micro-environment. On the other hand, active cues directly impose mechanical stimulations to which the cells similarly need to respond. In the context of vessels, these mechanical stimulations typically come in the form of hemodynamic loading. In the following subsections, we will briefly discuss the roles of passive and active cues in guiding cell response. We will specifically focus on the phenomenology of cellular mechanosensing that can offer direct inputs for designing instructive TE scaffolds. For detailed insights into the molecular pathways underlying these mechanosensing and mechanotransduction processes, we refer the reader to excellent dedicated reviews [53,54,55].

### 3.1. Passive Cues: The Cellular Micro-Environment

**Figure 4:**
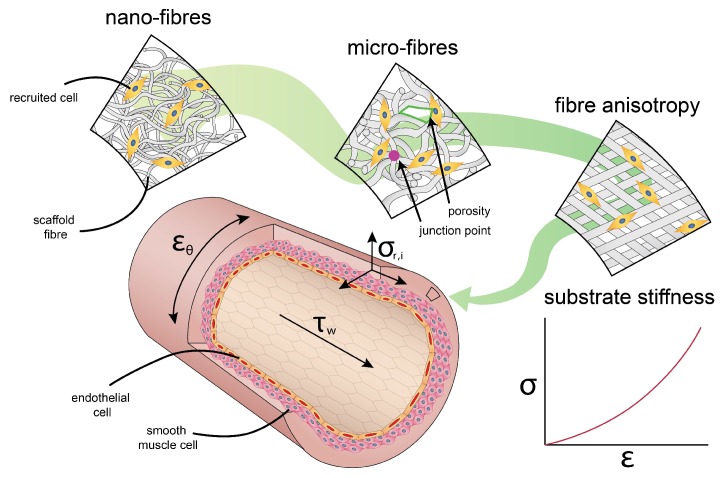
Passive and active cues in the context of scaffold design parameters and cardiovascular systems. Passive cues define the physical environment in which the cells reside, such as fibre diameter (nano-fibres vs. micro-fibres), topography (isotropic vs. anisotropic), and substrate stiffness. Active cues directly impose mechanical stimulations to the cell, such as shear stress ($\tau_{w}$), cyclic strain ($\varepsilon_{\theta }$), and residual stress ($\sigma_{r,i}$).

The recent emergence of the importance of passive cues has been spurred by the realization that tissues or scaffolds not only serve as physical structures that simply hold cells in place, but can also be used as instructive platforms that guide cell behavior. Importantly, this observation is made possible through systematic in vitro experimentations that present cells with well-defined and tunable cellular environments, allowing delineation of individual physical factors that are often convoluted in conventional in vivo situations. Among the various passive cues that have been identified, dimension, topography, and stiffness are probably the most well-studied and relevant for our current study (Figure 4).

#### 3.1.1. Dimension

All cells in the human body reside in a three-dimensional (3D) environment with diverse microstructural features. In contrast, traditional cell biological experiments have been mostly performed on two-dimensional (2D) culture plates. Cells grown on 2D surfaces are forced to assume apical–basal polarity and are usually flat with surface contact area that is much larger than their 3D cross-sectional area, because they can adhere and spread freely on the planar surface but have no support in the vertical direction. Such morphology is unnatural for most vascular cells responsible for tissue maintenance and remodeling (e.g., fibroblasts, smooth muscle cells), which are typified by a stellate morphology in 3D, and may (artificially) induce αSMA stress fibre expression [56]. Furthermore, at the length scale of individual cells (μm), cells often encounter essentially 1D continuous substrates, in such forms as scaffold microfibres or native collagen fibres. These 1D substrates form readily identifiable tracks that guide cell attachment, alignment, and migration. The exact molecular mechanisms that endow cells with the ability to sense (and respond to) the dimensionality of the substrate remains to be discovered.

#### 3.1.2. Topography and Spatial Distribution of Substrate Ligands

From a structural point of view, the extracellular matrix offers a diverse microarchitecture for the cell: for example, continuous substrate on sheet-like basement membrane, discontinuous environment in fibrous extracellular matrix (ECM) and scaffolds, and junction points between fibres. This diverse microarchitecture presents various topographical challenges that the cells need to deal with and navigate through.

In 3D ECM, pores within the matrix can create a confinement effect for the embedded cells. This steric confinement effect puts a physical limit to the cells’ mechanoresponse and therefore can play a particularly important role in situations where cells are induced, through other mechanisms such as mechanical straining or chemoattraction, to reorient or migrate. For example, cells have been shown to reorient perpendicular to the direction of cyclic straining in matrices with large pores—a phenomenon called strain avoidance [57]—but are unable to do so in matrices with small pores [58]. This geometry-guided cell behavior can crucially determine the physiological function of tissues where mechanical loading and cellular organization play an important role, such as the heart and vessels. Furthermore, it has been shown that contractility-dependent mesenchymal cell migration is very much limited in dense collagen gels because the cells have to negotiate through constricting pores [59], where they need to rely much more on matrix metalloproteinase (MMP)-dependent enzymatic degradation of the ECM to make their way [60,61,62]. This also significantly affects the efficacy of drugs that target specific pathways in the contractility or ECM degradation machinery [61].

Moreover, the substrate-bound ligands that are responsible for cellular adhesion can be non-uniformly distributed within the scaffold/tissue. Cells respond to this non-uniformity by migrating up the gradient of ligands in a phenomenon termed haptotaxis. For example, endothelial cells have been reported to exploit haptotaxis in their migration patterns during angiogenesis and vasculogenesis [63].

#### 3.1.3. Substrate Stiffness

In addition to structural properties, mechanical properties of the environment also play an important role in governing cell behavior. The stiffness of the substrate has been shown to direct the differentiation of mesenchymal stem cells [64]. Importantly, this lineage commitment is myosin-dependent [64], indicating that active probing of the substrate stiffness through actomyosin contractility is necessary. Multiple mechanosensors have been proposed to be responsible for detecting substrate stiffness, including local mechanosensing by focal adhesions [65] and global mechanosensing by the cytoskeleton [66,67]. Indeed, the expression and organization of actin stress fibres, the primary force-generation element in the cell, are also affected by substrate stiffness. Note that substrate stiffness and ligand presentation can work together to regulate the number and maturation of focal adhesions, and thereby also stress fibres and other downstream processes [62,68,69]. To delineate the role of substrate stiffness, therefore, it is important to make sure that the chemical and mechanical stabilities as well as the surface abundance of the ligands are kept constant. In most in situ TE applications, scaffold stiffness and functionalization can usually be designed independently from each other.

Sensing of substrate stiffness also manifests itself in the directional migration of cells up the stiffness gradient—termed durotaxis. In their pioneering study, Lo et al. showed that, on polyacrylamide substrates with a stiffness range as small as 14–30 Pa, fibroblasts can sense the substrate stiffness and migrate towards stiffer regions [70]. This study has since been reproduced using various cell types and stiffness ranges. Durotaxis has been proposed to play a role in atherosclerosis [71], where the local tissue mechanical properties in the atherosclerotic lesions are altered [72]. Indeed, durotaxis implies that cells can detect not only the global stiffness of the substrate, but also the local (subcellular) substrate stiffness. Local changes in mechanosensing can polarize the cytoskeleton and promote directional cell migration. This raises the question of how deep (or far away) a cell can sense the mechanical properties of its micro-environment [73]. Recently, it has even been shown that stem cell differentiation is affected by the local, not global, stiffness of the substrate, especially on native-ECM-like fibrous matrices [74,75].

### 3.2. Active Cues: Hemodynamic Loading

In contrast to mechanical cues that are ‘passively’ provided by the cell’s micro-environment, active cues coordinating cell behavior are a result of the dynamic environment in which these cells reside. For example, the dynamic nature of the cardiovascular system, consisting of the heart, blood, and blood vessels, and the response of its vascular cells to hemodynamic loads is reflected in the tissue architecture of the vascular wall [76] (Figure 4).

As a result of the pulsatile blood pressure, generated by the beating heart, the vascular wall is continuously subjected to *cyclic circumferential stresses* of around 100–150 kPa, resulting in typical strains of 10–15% [49]. The blood that circulates through the vessels exerts an *oscillatory shear stress* on the vascular wall of around 1–5 Pa (in humans) which varies with the exact location in the arterial tree. These values also significantly differ between different species, with a general trend of decreased mean shear stress with increased body size [77,78]. Such differences imply that endothelial cells (ECs) are ‘primed’ to different magnitudes of shear stress. Indeed, the exact set point is found to be mediated by VEGFR3, a signalling protein involved in shear stress sensing [79]. Although typical shear stresses are 5 orders of magnitude lower than circumferential stresses, its importance on cell behavior should not be underestimated as we will discuss in the following paragraph. Finally, due to pre-strained intramural elastin, medium to large scale arteries exhibit significant *residual stresses*, including axial stress. These values are not directly measurable in vivo, but values for pre-stretch can easily be estimated from explanted vessels, differing between species and with location from 1.5 to 1.7 [80]. Residual stresses originate from somatic growth, where elastin maturation occurs before the blood vessel reaches its final diameter [80,81].

The development of residual stresses depends on the relative rates of tissue growth and tissue maturation, and thus occurs in later stages of the in situ TE process. On the other hand, scaffolds will directly be exposed to blood hemodynamics upon implantation. To understand how blood pressure- and flow-related biomechanical forces influence cell-mediated tissue growth and remodeling, we require an intermediate step that translates classical biomechanics into single-cell mechanics. In this translation step, the micro-environment around the cell is a crucial mediator.

#### 3.2.1. Shear Stress

Shear stress affects both intracellular and intercellular behaviour. To this mechanical cue, the ECs are directly exposed, as they form the thin interface between the blood and the rest of the vascular wall. Shear-stress-dependent EC responses, including shape, alignment, monolayer formation, and intercellular interactions with other vascular cells, have been demonstrated in in vitro 2D studies [82,83,84,85]. A number of these responses, e.g., cellular alignment, are found to be mediated via VEGFR2 [86]. Due to its nonthrombogenic properties, endothelium at the scaffold wall is the ideal blood-contacting surface, reducing the risk of thrombus formation. For this purpose, one of the strategies is the regeneration of an endothelial layer, which relies on recruitment of endothelial progenitor cells (EPCs) from the bloodstream [87]. After cell capture, the scaffold’s microarchitecture dictates the capacity of ECs to form an impermeable boundary. For example, fibre diameter has been shown to be an important parameter for monolayer formation [88].

Before endothelialization occurs, the interaction between biomaterial and blood provokes a foreign body response. This initial response includes protein absorption to and provisional matrix formation in and around the biomaterial [89]. The provisional matrix (i.e., blood clot) prevents fibrous scaffolds from leaking and attracts other cells to the biomaterial. To prevent random adhesion of cells, which often leads to intimal hyperplasia on the long term, efforts are focused on creating non-cell adhesive surfaces which can be further functionalized to recruit specific cell populations, such as EPCs [90].

Other cell types that reside in the vascular wall are smooth muscle cells (SMCs) and fibroblasts (FBs), which are located in the media and adventitia, respectively. Interestingly, ECs, SMCs, and FBs do not respond to mechanical cues in the same way. In vitro 2D flow experiments show that, whereas ECs align parallel to the flow direction, SMCs orient themselves perpendicularly to perfusion [91]. In 3D, the transmural pressure gradient causes SMCs and FBs to be exposed to transmural shear stresses, typically of 1 order of magnitude lower than luminal shear stresses, i.e., 0.1 Pa [92]. Despite the relatively small magnitude, shear stress has been shown to strongly influence the signaling, proliferation, contraction, and phenotype of these cells, as extensively reviewed elsewhere [93].

The above described cellular responses to wall shear stress have important implications for the design of scaffolds. For example, wall shear stress at the luminal wall is inversely proportional to the third power of the internal radius. This emphasizes the importance of matching the scaffold geometry to its final application, which is dictated by the location where a graft is needed [94]. In addition, the scaffold’s pore size affects transmural flows, with smaller pores resulting in higher transmural shear stresses [92,95]. From this perspective, pore size is an important design parameter not only for controlling cellular infiltration, but also for the application of mechanical cues.

#### 3.2.2. Cyclic Stress (and Strain)

Since the cardiovascular system is pressure-driven, the degree of circumferential stress in the vascular wall depends on the blood pressure and the wall-thickness-to-diameter-ratio via the well-known Laplace law. How these stresses result in strains is in turn dictated by the scaffold’s macroscopic mechanical properties. Such macroscopic strains determine the blood distribution within the circulation, but are not representative of what the cells feel locally [96]. Instead, cells most likely sense the *local* stresses (or strains) in the substrate to which they adhere (Section 3.1). The fibre organization (such as orientation, interconnections, porosity, and fibre diameter [97]), the substrate’s fibre stiffness, and cell adhesion to the fibre determine the magnitude of transmitted macroscopic forces. This emphasizes the relevance of tuning the scaffold’s micro-environment and substrate stiffness.

Although it remains unclear whether strain-induced stress or stress-induced strain is the mechanical trigger for cell-mediated tissue growth and remodeling, the fact that mechanical forces influence growth rate and direction is well-known [81]. Growth, defined as an increase in cell volume, cell number, and/or ECM, is promoted with stretch but inhibited by compression [98]. Tissue growth and cell alignment occur along the constraint direction if statically applied [99], but along the direction of minimal deformation rate if cyclically applied and in confined situations [100,101]. The cellular behaviour in response to cyclic deformations is referred to as strain-avoidance. In this process, the Rho pathway is identified as a key regulator [102,103]. The combination of cyclic stretch with shear stress is physiologically relevant, if perpendicularly applied to one another, and has been shown to reinforce EC alignment along the flow direction [104].

Despite what was noted earlier, mechanical triggers alone cannot explain the observed cellular organization of SMCs in the vessel wall: even though the vessel wall is mostly cyclically stretched in the circumferential direction, SMCs are mainly circumferentially oriented as well. This cellular alignment allows the vessel wall to efficiently contract in response to circumferential loading [76]. The explanation for this unexpected cellular organization is generally found in the guidance effect of the ECM: cells not only respond to mechanical triggers, the ECM also provides structural triggers for directional growth and orientation (see Section 3.1). Stretch-induced growth and remodeling can be overruled by contact guidance provided by the ECM, as has been shown in vitro [101,105]. This mechanism makes the circumferentially aligned collagen fibres in the vessel wall a guidance for cell orientation in vivo—a strategy that we can adopt when designing scaffolds for in situ TE.

#### 3.2.3. Residual Stress

Residual stresses refer to stresses that are still present in the absence of actively applied loads. Different degrees of residual stress are manifested in different degrees of pre-strain, which is proportional to the opening angle and length shortening after blood vessel explantation. Similar to cyclic strains, pre-strains also exist across multiple spatial scales. The pre-strain at the cellular level, due to active cell contraction and residual stresses in the ECM, plays an important role in the macroscopic behavior of blood vessels and heart valves [106].

The role of pre-strain on macroscopic arterial wall mechanics, i.e., tissue stiffness, finds its origin in nonlinear continuum mechanics (‘strain-stiffening’): soft biological materials become stiffer at large deformations. This way, tissue integrity is protected at large pressures. When strain-stiffening materials are pre-strained, their apparent material stiffness decreases [107,108]. In other words, the right amount of pre-strain allows the vessel wall to position itself in its optimal operating range, which is hypothesized to be the transition point where strain-stiffening behavior starts [107]. The importance of this principle, modulation of apparent material stiffness by modulation of residual stress, becomes clear from pathological cases, such as altered ECM or hemodynamic loading. Axial pre-strain is able to compensate for such environmental changes, ensuring a similar circumferential stress-stretch response among healthy and diseased cases, as excellently illustrated in more detail elsewhere [80].

Such protective, adaptive properties of soft tissue are of vital importance to ensure long-term functionality of in situ TE blood vessels and heart valves. The design of the scaffold should therefore allow for the development and adaptation of residual stresses. In general, such stresses already naturally develop in in vitro tissue-engineered vessels, and they are further enhanced with mechanical loading. Similar to native arteries and valves, elastin plays a crucial role in the development of these residual stresses [109]. Therefore, strategies to promote elastin formation and maturation are important to incorporate in the development of instructive scaffolds, especially for applications under arterial pressures.

## 4. Towards a Hypothesis-Driven Engineering Approach

In the previous sections, we have reviewed some of the most relevant physiological cues that guide cellular response and thus play a determining role in the development and maturation of tissues. As such, detailed insights into cellular mechanobiology offers promising new strategies for designing scaffold constructs for regenerative medicine, especially for in situ tissue engineering applications, by exploiting the instructive properties of the passive and active environmental cues. Furthermore, we have highlighted that these cues are highly dynamic and can simultaneously influence each other. To aim at a hypothesis-driven approach in regenerative medicine, therefore, there is a clear need for experimental platforms that allow delineation of the role of each factor, both in terms of passive and active cues, in a controlled and systematic manner. Moreover, as we have also discussed, in virtually all physiological settings, *multiple* passive and active environmental cues act on the cells at any given time. The combinatorial effects of these multiple cues are still poorly understood, primarily because of the technical difficulty in developing experimental platforms that allow full independent control over multiple cues. In the following sections, we describe various microfabricated systems, scaffolds, and bioreactors that have provided invaluable insights into the individual factors. Finally, new opportunities for tissue-engineered vessels (TEVs) are discussed.

### 4.1. Passive Mechanostimulation of Cells

The vascular topographical environment is macroscopically formed by the large collagen fibre bundles that constitute the ECM of vessel walls. The collagen fibrils themselves and the associated proteins form a microscopic (or even nanoscopic) topographical micro-environment. Studying the role of topography and ligand distribution at the nm to μm length scales on cell function and the underlying molecular mechanisms using microgrooves and micropatterning approaches has yielded important insights on cell function and the underlying molecular mechanisms (see detailed reviews by [110,111,112,113], Table 3). Recent studies that present multiple mechanical and multi-scale topographical cues to the cells indicate that these cues interestingly can cooperate and compete to direct cellular response [114,115,116,117]. Micropatterning methods have been further optimized to reach nanometer-scale patterning by employing protein nanodots [118].

Another type of passive mechanostimulation is presenting cells to different substrate stiffnesses. Substrate stiffness in 2D has been revealed to govern various cell functions, including morphology, adhesion, stiffness, differentiation, and migration [64,69,129,130,131,132] (Table 3), which in turn affect tissue formation and maturation [133]. However, it is important to note that the initial, bulk mechanical properties of the scaffold are often not sufficient to predict cellular behavior, especially at time scales of days or longer. First, since cells are able to actively remodel the surrounding micro-environment, the tissue mechanical properties do not stay constant [134,135]. In fact, a change in tissue stiffness is often a good prognostic indicator for various diseases [136], for example vessel wall stiffness in atherosclerosis [137] and cardiovascular death [138]. Second, how the global mechanical properties of the scaffold translate to local mechanical properties, and hence also how large-scale active cues (e.g., mechanical loading) to the scaffold are transmitted to individual cells, is surprisingly difficult to predict [139,140], since it mainly depends on the exact microarchitecture and connectivity of the fibres within the scaffold [141,142]. Third, the properties of the ECM can themselves change over time, even in the absence of cells, due to its response to mechanical loading. Cell-free fibrin and collagen fibre networks have both been shown to undergo weakening under cyclic loading [143,144]. This weakening is furthermore accompanied by the appearance of residual strains that progressively grow not only with repeated loadings but also with increasing loading amplitude, due to internal remodeling at the network and fibre scales [145].

In light of these complexities, recent efforts have focused on the quantification of spatiotemporally-resolved local mechanical properties of hydrogels, enabling the study of substrate stiffness in 3D. Interestingly, studies using different types of hydrogels yielded opposing results: cells locally stiffen collagen gels by bundling together and recruiting collagen fibres [146], but fluidize PEG gels [147], suggesting that cells can adaptively ‘prime’ their surrounding micro-environment depending on the local microstructure and mechanical properties. An aspect that has been under-investigated in the studies using 3D gels is structural and mechanical anisotropy. This is especially important considering that most biological tissues, including the heart and vessel wall, exhibit well-defined organization and aligned ECM and cells. Since creating hydrogels with anisotropic structure is tricky, a more promising step to investigate the role of structural and mechanical anisotropy is by using scaffolds produced using electrospinning, fused-deposition modeling, or 3D printing, where the extent of anisotropy can be more directly achieved and controlled [148].

### 4.2. Active Mechanostimulation of Cells

Cell cultures on flat substrates, introduced more than a century ago [149], form an intrinsic part of most research laboratories nowadays. Although these 2D cultures have now become more complex and physiologically relevant by combining multiple cell types and cell patterning (Section 4.1), linking such cultures to the in vivo situation remains challenging. In an attempt to better understand cell behaviour in its physiological environment, numerous studies added mechanical stimulation to their 2D cell cultures. Cardiovascular related cells, i.e., ECs, SMCs, and FBs, have been exposed to shear stress, cyclic stretch, or a combination of the two (Table 3). However, none of these methods take into account the cell’s micro-environment. This means that interactions between cells and the surrounding matrix, being not only a source of passive cues, but also a transmitter of active cues, are difficult to study using such platforms. In addition, due to the structure-function relation, the primary vessel function arises at a macroscopic level, which cannot be captured using these platforms (Figure 2). Therefore, testing scaffolds for in situ TE requires in vitro platforms where the cell-scaffold construct as a whole can be exposed to physiologically-relevant mechanical stimuli.

### 4.3. Combined Methods

In addition to their clinical appeal, TEVs are generated to gain more insight into (the combination of) active and passive mechanostimulation of cells. To this end, providing the right environment, in terms of temperature, pH, biochemical signals, electrical signals, and mechanical signals, becomes relevant. The right environment can be provided by a bioreactor, which typically consists of four components: a culture chamber, a motor-driven pump, a medium reservoir and a temperature controller [150]. Bioreactors also offer great possibilities for testing biodegradable scaffolds. Similar to 2D mechanostimulation for cardiovascular purposes, mechanical loading in 3D usually focuses on stretch, shear stress, and a combination of the two (Table 3).

With the current advances in the field, a new motivation to engineer blood vessels in vitro appears: they can be used as 3D in vitro devices to model disease and to develop and test drugs [151]. For example, TEVs can be used to test the deployment of intravascular devices (e.g., stents). Using these models, not only the damage to the confluent layer of ECs, as a result of device placement, can be modeled, but the consequences of this damage to other vascular cells (i.e., SMCs) can be simulated as well. Another example is to use TEVs as a model for atherosclerosis, i.e., the deposition of fatty material on the inner vascular wall. Here, the effect of flow-induced shear stress and cyclic strain on the progression of the disease is very important. A bioreactor capable of mimicking these active cues is therefore an absolute requisite.

## 5. Concluding Remarks and Outlook

**Figure 5:**
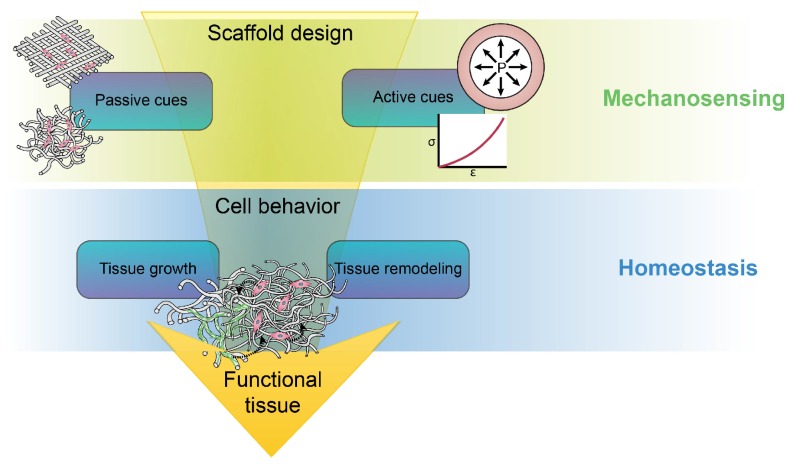
A rational design of a scaffold should provide the right passive cues and transmit the right active cues to guide cells towards a mechanical homeostasis via growth and remodeling. The process of growth and remodeling is both active (cell-mediated) and passive (e.g., dilatation of the vessel wall and micro-structure dictated by degree of axial pre-stress). Following this approach results in controlled in situ regeneration.

In vivo regeneration of tissues and organs always obeys the rules (or at least the physical guidance) set by the cellular micro-environment. To have a better control of in situ tissue regeneration, therefore, it is unavoidable that one should make careful decisions about the micro-environment to which cells are dynamically exposed, mainly in terms of the passive and active cues as well as their combinations, which we have described (Figure 5). To be able to make these decisions, two complementary intermediate steps need to be taken: (i) we need to understand how specific cellular responses are governed by the individual environmental cues, which should lead to predictive mechanobiological models for tissue formation and maturation, and (ii) we need to directly test these predictions using experimental platforms that can closely recapitulate the (patho)physiological micro-environment. As these two fronts are making progress and advanced multi-stimuli bioreactors are being developed, we anticipate a progressive shift towards a rational and effective control of in situ vascular tissue regeneration. This offers exciting new prospects not only for the regeneration of relatively thick-walled blood vessels that we described here, but also for extrapolating the principles to endothelium-dominated mechanobiology in the small microvasculatures [152] and to lymphatic vessels [153,154].

## Figures and Tables

**Table 1:** Mechanical properties, measured in terms of compliance and burst pressure, and structural properties, measured in terms of wall thickness, inner diameter, and vessel wall thickness-to-radius (T:R) ratio, from a selection of studies concerning native tissue, synthetic grafts, in vitro tissue-engineered grafts, and in situ tissue-engineered grafts. Compliance is measured in the physiological range, i.e., 80–120 mmHg, unless indicated otherwise.

Artery	Material	Design	Model	Compliance (%/100 mmHg)	Burst Pressure (mmHg)	Thickness (μm)	Inner Diameter (mm)	T:R (-)	Ref.
Native	AA	n.a.	rat	6.7	3415	150	1	0.3	[13]
	CA	n.a.	porcine	18.7 *	3320	614	n.d.	n.d.	[14]
	IMA	n.a.	porcine	11.2	2100	231	n.d.	n.d.	[15]
	FA	n.a.	sheep	3.3	2297	770	n.d.	n.d.	[16]
	FA	n.a.	sheep	8.52 ^†^	n.d.	n.d.	n.d.	n.d.	[17]
	CA	n.a.	sheep	11.98	10,950	750	2.25	0.67	[18]
	FA	n.a.	human	2.6	n.d.	n.d.	n.d.	n.d.	[19]
	IMA	n.a.	human	4.5–6.2	2031–4225	350–710	n.d.	n.d.	[9]
	IMA	n.a.	human	11.5	3196	300–800	1.5–4.5	0.35–0.40	[20]
Synthetic	PGA or PLLA + PLCL	non-woven porous graft	n.a.	n.d.	2710–2790	150–250	0.7–0.9	0.33–0.71	[21]
	PCL	e-spun microfibrous graft	n.a.	0.58–0.92	850–1800	415	6	0.14	[22]
	PLLA	e-spun microfibrous graft	n.a.	0.93	n.d.	390	4.9	0.16	[17]
	PLLA/PLCL	bi-layered graft with inner e-spun and outer weft-knitted layer	n.a.	1.8	21,750	330	3.2	0.21	[23]
	PLLA/PHD	bi-layered graft with different blend rations	n.a.	1.12	1775	230	5	0.09	[24]
	PEUU	bi-layered graft with large inner pores and dense outer pores	n.a.	4.6	2300	743	4.7	0.32	[15]
	poly(diol citrate)	non-woven porous graft	n.a.	12.7	250	160	3.65	0.09	[25]
In vitro	PGA	non-woven porous graft	10 weeks under pulsatile conditions	n.d.	1300–1337	442	3	0.29	[26]
	PGA	non-woven porous graft	1 week static, 4 weeks dynamic strain (1%)	n.d.	906	1000	3	0.67	[27]
	human fibroblast sheets	sheet-based	8 weeks static, 10 weeks maturation	1.5	3468	407	4.2	0.19	[9]
	fibrin	fibroblast-seeded fibrin gel	2 weeks static, 5–7 weeks dynamic strain (7%)	2.4–4.4	1366–1542	280–430	2–4	0.22–0.28	[16]
	PGA	non-woven porous graft	7–10 weeks dynamic strain (2.5%)	3.3	3337	1000	6	0.33	[8]
	PGA	non-woven porous graft	7–8 weeks dynamic strain (1.5%)	3.5 *	800	220	3	0.15	[14]
	human fibroblast sheets	sheet-based	6–8 weeks static, 12 weeks maturation	3.54	3490	200–600	2.4–6.6	0.18	[20]
In situ	PCL/CS	e-spun nanofibrous graft	sheep (CA), 6 months	6.58	10,275	1180	2.9	0.81	[18]
	PEOT/BPT/PCL	PEOT/BPT solid rod with external e-spun PCL sheet	porcine (SC), 4 weeks	7.46	3947	700	2	0.70	[10]
	PCL	e-spun nano/microfibrous graft	rat (AA), 1.5–18 months	7.8	3280	650	2	0.65	[28]
	PGS/PCL	porous PGS reinforced with PCL sheet	rat (AA), 3 months	11	2360	290	0.72	0.81	[13]

AA, abdominal artery; CA, carotid artery; FA, femoral artery; IMA, internal mammary artery; PGA, polyglycolic acid; PLLA,poly-L-lactic acid; PLCL, 50:50 copolymer solution of L-lactide and ε-caprolactone; PCL, poly(caprolactone); PEUU, poly(esterure-thane)urea; CS, chitosan; PEOT/BPT, poly(ethylene oxide terephthalate)-poly(butylene terephthalate; SC, subcutaneous; PGS, poly(glycerol sebacate); n.a., not applicable; n.d., not determined; *, 80–200 mmHg; ^†^, 60–190 mmHg.

**Table 2:** Selection of in vivo studies applying the in situ TE approach, with a specific focus on long-term functionality.

In-Vivo Model	Material	Design	Implantation Time	Main Outcome	Ref.
human	PLCL/PGA or PLLA	knitted PGA or PLLA fibers with PLCL sponge *	4.3–7.3 years	no graft related deaths, TEVGs are technically feasible	[11]
mouse	PGS/PCL	microfibrous PGS core with PCL outer sheet	12 months	perfect patency, progressive luminal enlargement due to PGS degradation	[47]
mouse	PGA/PLCL	non-woven porous graft with outer PLCL sheet	24 months	biomechanical diversity among implanted vascular grafts due to variations in the ratio collagen type I/III	[46]
rat	PCL	e-spun nano/microfibrous graft	18 months	perfect patency with excellent structural integrity, but calcifications appeared in the IH layers	[28]
mouse	PLCL/PLA	non-woven porous graft	12 months	well-organized neotissue formation, but mos mice developed aneurysms	[29]
dog	PGA/PLCL/ P(GA-CL)	knitted PGA fibres with PLCL sponge and outer P(GA/CL) reinforcement	12 months	no aneurysmal change or stenosis, but underdeveloped VSMCs	[48]

PLCL, copolymer solution of L-lactide and ε-caprolactone; PGA, polyglycolic acid; PLLA, poly-L-lactic acid; TEVG, tissue-engineered vascular graft; PGS, poly(glycerol sebacate); PCL, polycaprolactone; PLA, poly-lactic acid; P(GA-CL), copolymer solution of glycolic acid and ε-caprolactone; *, pre-seeded with autologous mononuclear cells.

**Table 3:** Selection of currently available experimental platforms that are used to delineate the role of passive and active cues in the context of optimizing key scaffold properties.

Key Scaffold Properties	Mechanostimulation	Technique to Study	Variables	Current Limitations
	*Passive*			
fibre diameter, fibre topography		• microgrooves	• groove width (nm–μm) • shape	groove-depth as confounding parameter
	• dimension/topography			
		• micropatterning	• pattern size (μm) • shape • protein gradients	range of pattern-size
fibre stiffness, macroscopic stiffness, scaffold density	• substrate stiffness	• polyacrylamide gels (2D) [119]	• 1 Pa–100 kPa	unable to capture fibrous 3D morphology
		• hydrogels (3D) [120,121]	• <1 Pa–few kPa • stiffness gradients (2D) • non-linearity	low stiffness magnitude
	*Active*			
anisotropy, geometry	• shear stress	• parallel plates [122]	• shear stress (<1 Pa–few Pa)	pressure as confounding parameter
		• orbital shaker [122]	• shear stress (<1 Pa–few Pa)	temporal and spatial variations in shear stress
anisotropy, geometry, macroscopic stiffness	• strain	• motor/pressure driven distensible membrane [122]	• strain (1–20%)	spatial variations in strain
anisotropy, geometry, macroscopic stiffness	• shear stress & strain	• mock artery [122]	• shear stress (<1 Pa) • strain (1–10%)	no independent control of variables
		• microfluidic device [123,124,125]	• shear stress (<1 Pa–few Pa) • strain (1–10%)	lack of 3D environment
	*Passive and active*			
fibre diameter, anisotropy, pore size	• scaffold + shear stress	• parallel plates in mesofluidic device [126]	• shear stress (<1 Pa–few Pa) • scaffold properties	pressure as confounding parameter
anisotropy, pore size, connectivity, macroscopicstiffness, degradation rate	• scaffold + strain	• motor/pressure driven distensible membrane [99,127]	• strain (1–20%) • scaffold properties	spatial variations in strain
fibre diameter, anisotropy, pore size, connectivity, macroscopic stiffness, degradation rate	• scaffold + shear stress & strain	• perfusion bioreactor [128]	• shear stress (<1 Pa–few Pa) • strain (1–5%) • scaffold properties	no independent control of active variables
